# The Prevalence and Risk Factors of Hyperkalemia in the Outpatient Setting

**DOI:** 10.1155/2024/5694131

**Published:** 2024-01-22

**Authors:** Chadapa Sevamontree, Supreeya Jintajirapan, Pran Phakdeekitcharoen, Bunyong Phakdeekitcharoen

**Affiliations:** ^1^Division of Nephrology, Department of Medicine, Faculty of Medicine, Ramathibodi Hospital, Mahidol University, Bangkok, Thailand; ^2^Outpatient Intervention and Urgency Care, Department of Nursing, Faculty of Medicine Ramathibodi Hospital, Mahidol University, Bangkok, Thailand; ^3^Renal Department, Sunderland Royal Hospital, Sunderland, UK

## Abstract

**Background:**

Hyperkalemia is a life-threatening condition in outpatient and emergency departments. Hyperkalemia is associated with more events of major adverse cardiovascular diseases, hospitalization, and death. The aim of this study is to study and assess the prevalence and risk factors for developing hyperkalemia within the Thai population.

**Method:**

A cross-sectional observational study of 3,299 unique adult patients (≥18 years) in one calendar year (2021) with at least 1 valid serum potassium (SK) test was conducted in the outpatient department of medicine. Hyperkalemia was determined as SK ≥5.8 mmol/L without hemolysis or technical error. Clinical data and laboratory tests were collected for analysis of risk factors.

**Result:**

2,971 patients (131 hyperkalemia and 2,840 control) were eligible. The annual prevalence of hyperkalemia was 4.41%. The mean ages of patients were 66.5 years in the hyperkalemia group and 55.9 years in the control group. Increasing age had a positive association (*r* = 0.220, *p* < 0.001) to risk of hyperkalemia, whereas the estimated glomerular filtration rate (eGFR) had an inverse association with SK level (*r* = −0.398, *p* < 0.001). The risk factors for hyperkalemia were patients with age ≥65 years (odds ratio, 2.106; 95% CI, 1.399, 3.171; *p* < 0.001), presence of diabetes mellitus (DM, odds ratio, 1.541; 95% CI, 1.030, 2.306; *p* = 0.036), chronic kidney disease (CKD) stage ≥3 (odds ratio, 14.885; 95% CI, 8.112, 27.313; *p* < 0.001), hemodialysis treatment (odds ratio, 10.170; 95% CI, 5.858, 17.657; *p* < 0.001), and usage of renin-angiotensin-aldosterone system inhibitors (RAASi, odds ratio, 2.256; 95% CI, 1.440, 3.536; *p* < 0.001).

**Conclusion:**

The risk factors contributing to hyperkalemia were patients with older age, DM, CKD, hemodialysis treatment, and usage of RAASi. Although the usage of RAASi is proven to be a cardiovascular advantage in the elderly, DM, and CKD patients, careful monitoring of SK is strongly advised to optimize patient care.

## 1. Introduction

Potassium is the most abundant cation in the intracellular fluid and plays an important role in regulating cell function. This is particularly the case for excitable tissues such as nerve, cardiac, and skeletal muscle, where sodium, potassium, and calcium are the main ions that control the action potential of the cells. Potassium concentration in the extracellular fluid is pivotal for setting the resting membrane potential of the cells and must be confined within a tight range, between 3.5 and 5.0 mmol/L [1]. The kidney is the principal organ responsible for maintaining total body potassium abundance by balancing potassium reabsorption with excretion [2].

Hyperkalemia is defined as a serum potassium concentration exceeding 5.0 mmol/L. It is often categorized as mild (>5.0–5.9 mmol/L), moderate (6.0–6.4 mmol/L), and severe (≥6.5 mmol/L) [3]. Severe hyperkalemia is a life-threatening condition associated with severe morbidities and mortalities. Severe hyperkalemia is a medical emergency since it can impact the cardiovascular system. Hyperkalemia can cause cardiac arrhythmias including sinus bradycardia, sinus arrest, slow idioventricular rhythms, ventricular tachycardia, ventricular fibrillation, and asystole [4]. Fatal complications are associated with major adverse cardiovascular events, hospitalization, and higher rates of death [5, 6]. Moreover, hyperkalemia is associated with a remarkable economic burden on affected individuals and the medical care system [7].

The primary control for potassium balance is the renal excretion mechanism, which regulates the amount of potassium excreted or reabsorbed from the kidneys. Important influences of potassium excretion are factors that affect potassium secretion along the distal nephron [2]. Hyperkalemia has a direct effect on the adrenal cortex and stimulates aldosterone secretion [8]. Aldosterone plays an important role in controlling potassium excretion in the collecting tubules of the kidneys. Hyperkalemia can primarily be caused by transcellular potassium shift and inadequate renal excretion. Several factors can cause transcellular potassium shifts, such as acidosis, insulin, *ß*-adrenergic agonists, thyroid hormones, aldosterone, and exercise [9–12]. However, common causes of hyperkalemia result from inadequate renal potassium excretion; this can be classified into two groups—impairment of renal function and disruption of the renin-angiotensin-aldosterone system (RAAS). In the former group, a reduction in renal mass and glomerular filtration rate (GFR), e.g., in chronic kidney disease (CKD) patients, can result in a reduction in potassium excretion, leading to an accumulation of potassium in the body. In the latter group, the renal tubular excretion of potassium can be compromised through disruption of RAAS, e.g., medications such as angiotensin-converting enzyme inhibitors (ACE inhibitors), or tubular resistance to the action of aldosterone (e.g. renal tubular acidosis), leading to altered potassium excretion and subsequently hyperkalemia [2, 13].

The prevalence of hyperkalemia in the US population was 1.57% in 2014, where a high frequency of these patients also had CKD, heart failure, diabetes mellitus and/or hypertension [14], and among CKD patients using renin-angiotensin-aldosterone system inhibitors (RAASi) [15]. In a systematic and meta-analysis review (by any definition or threshold), the prevalence of adult hyperkalemia (age ≥ 18 years) and hyperkalemia in outpatient or primary care settings was 6.3% and 5%, respectively, and the incidence of hyperkalemia in the adult population was 2.8 cases per 100 person-years [16].

There are insufficient data on the epidemiology and comorbidity of hyperkalemia in non-Western populations, especially in the Thai population. Our studies, therefore, aim to evaluate the prevalence of hyperkalemia in the outpatient unit of the Department of Medicine in one of the largest university hospitals in Thailand. The secondary objective is to study the association between hyperkalemia and its risk factors in our population.

## 2. Methods

### 2.1. Study Population

A cross-sectional observational study was conducted on patients who visited the outpatient department of Medicine, Ramathibodi Hospital, Mahidol University, Bangkok, Thailand, from January 1st to December 31st, 2021, with serum potassium ≥5.8 mmol/L. We chose a cut-off serum potassium of 5.8 mmol/L as this was the hospital's protocol—any value ≥5.8 mmol/L would trigger an urgent response from the laboratory to the responsible physicians.

In the hyperkalemic group, all patients were included if all the following conditions were present: (1) age > 18 years old, (2) serum potassium ≥ 5.8 mmol/L with an additional second blood sample for confirmation of hyperkalemia, and (3) consent from the patient to be enrolled. Exclusion criteria included (1) a lab report of hemolysis of blood samples or (2) inconsistent information. For patients that have multiple episodes of hyperkalemia, one visit of hyperkalemia was randomly selected for the studies. In the nonhyperkalemic group, all registration data of patients who visited the outpatient department of Medicine with serum potassium <5.8 mmol/L and never had serum potassium more than or equal to 5.8 mmol/L in the calendar year were included. Also, in patients who have multiple visits of serum potassium <5.8 mmol/L, one visit of nonhyperkalemia was randomly selected for the studies. All methods were conducted according to the relevant guidelines and regulations of the institute. The study was approved by the Institutional Ethics Committee on Human Rights Related to Research Involving Human Subjects of the Ramathibodi Hospital, Mahidol University, Approval No. MURA2021/61.

### 2.2. Data and Sample Collection

Patients who visited the outpatient department of Medicine and had serum potassium of more than or equal to 5.8 mmol/L were notified for investigation and management in the outpatient intervention and urgency unit. Patient demographic details, comorbidities including diabetic mellitus, hypertension, coronary artery disease (CAD), CKD, on renal replacement therapy including hemodialysis (HD), continuous ambulatory peritoneal dialysis (CAPD), kidney transplantation, current medications, as well as other potential risk factors for developing hyperkalemia were recorded. Second serum potassium was collected for confirmation of hyperkalemia. Serum creatinine value was analyzed with an enzymatic method using an automatic device (Architect c16000, Abbott core laboratory, Illinois, USA). eGFR was calculated using the CKD-EPI equation (17).

The definition of CKD is the presence of kidney damage or decreased renal function for at least three months, irrespective of the cause. Kidney damage generally refers to pathologic anomalies in the native or transplanted kidney, established via imaging, biopsy, or deduced from clinical markers like increased albuminuria (albumin-to-creatinine ratio > 30 mg/g) or urinary sediment alterations; decreased renal function refers to a reduced eGFR <60 ml/min/1.73 m^2^ [18]. CKD stage 3a was defined as having an eGFR between 45 and 59 mL/min/1.73 m^2^, stage 3b has an eGFR between 30 and 44, stage 4 between 15 and 29, and stage 5 CKD <15 mL/min/1.73 m^2^. Dialysis mode was defined as its use within the last three months according to the local guidelines before the serum potassium test. Peritoneal dialysis was recorded as CAPD or automated peritoneal dialysis (APD).

Control subjects were patients who were more than 18 years old seen in the outpatient medical department and had all their serum potassium results less than 5.8 mmol/L in the calendar year. All clinical and laboratory data were recruited via chart review from the institute information collection center. The medical information for each patient visit was retrieved electronically from the Data Health for Analysis Informatics Section. The patient data were anonymized to maintain confidentiality. The abstracted data included age, demographics, previous medical history, laboratory results, and medications. A medical chart review was performed for all identified cases, including comorbidities, the medication used, underlying kidney disease, and mode of renal replacement therapy.

### 2.3. Statistical Analyses

The data were checked for normal distribution and data with a nonnormal distribution were log-transformed, after which normal distribution was confirmed before analysis. The parametric numeric data were presented as mean and standard deviation (SD), the nonparametric numeric data as the median and interquartile range (IQR), and the categorical data as number and percentage. The differences between parametric numeric data, nonparametric numeric data, and categorical data were tested by independent sample *t*-test, Mann–Whitney *U* test, and chi-squared test, respectively. Pearson's correlation coefficients (*r*) were used to examine the association among serum potassium levels, age, eGFR, and selected clinical variables. A multivariate linear regression model with the forward stepwise method was derived using variables with a *P* < 0.05 univariate correlation with serum potassium. We chose a cut-off *P* value of 0.05 since there were a sizeable number of significant variables in the univariate analysis, and we would like to avoid the model overfitting effect. Also, we chose the forward stepwise selection as the variables were biologically plausible and/or statistically significant. The results were presented with a standardized coefficient of beta (*ß*) and a 95% confidence interval for *ß* (95% CI). For categorical outcomes, a multivariate binary logistic regression model with the forward stepwise method was derived using variables with a *P* < 0.05 univariate correlation with hyperkalemia. The results were presented with an odds ratio (OR) and a 95% confidence interval for OR (95% CI). *P* value <0.05 was considered significant. All analyses were performed with IBM SPSS Statistics program version 23.0.

## 3. Results

### 3.1. Patient Characteristics

**Figure 1:**
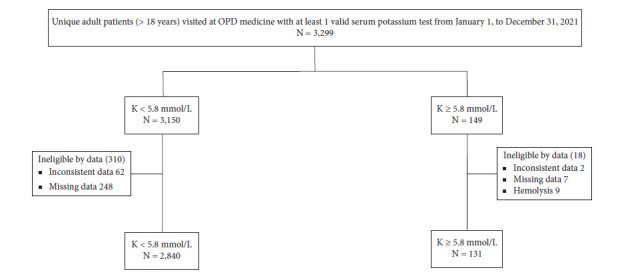
Flowchart showing patients included in the hyperkalemia and control groups in this study. OPD, outpatient department; *N*, number; K, serum potassium.

3,299 unique adult patients with at least 1 valid serum potassium test from January 1st to December 31st, 2021, were included, according to data visited in the outpatient department of medicine. This population was divided into 149 individuals with serum potassium ≥5.8 mmol/L and 3,150 individuals with serum potassium <5.8 mmol/L. In the hyperkalemia group (149 cases), 18 patients were excluded from the studies due to ineligible data including 2 cases of inconsistent information, 7 cases of missing data, and 9 cases of blood hemolysis. In the control group (3,150 cases), 310 patients were excluded from the studies due to ineligible data including 62 cases of inconsistent information and 248 cases of missing data. The final results contained 131 cases within the hyperkalemia group and 2,840 cases within the control group (Figure 1). The annual prevalence of hyperkalemia in this study was 4.41% in the year 2021.

Baseline characteristics of patients with serum potassium ≥5.8 mmol/L and <5.8 mmol/L in the study are summarized in Table 1. The mean serum potassium concentrations in the patients with hyperkalemia and control groups were 6.10 ± 0.28 and 4.24 ± 0.42 mmol/L, respectively. The mean age of patients with hyperkalemia was significantly higher than the control group (66.5 ± 13.4 vs. 55.9 ± 12.9 years). The median eGFR was significantly lower in the hyperkalemia than in the control group (16.9 vs. 78.3, mL/min/1.73 m^2^). Patients with hyperkalemia had significantly higher comorbidities including hypertension (69.5% vs. 58.7%), diabetes mellitus (55.0% vs. 36.3%), and history of CAD (28.2% vs. 15.7%) than the control group. Additionally, hyperkalemic patients had significantly higher coexisting conditions such as CKD stage ≥3 (90.1% vs. 25.8%), hemodialysis (26.0% vs. 1.44%), CAPD (3.05% vs. 0.18%), and kidney transplants (5.34% vs. 1.44%) than the control group. The medications that were more frequently found in patients with hyperkalemia than the control group in this study were RAASi (29.0%, vs. 15.4%), including angiotensin receptor blocker (ARB, 17.6% vs. 10.7%) and mineralocorticoid receptor antagonist (MRA, 9.92% vs. 2.01%) (Table 1). There were 39 patients in this study using multiple RAASi: 5 out of 131 (3.82%) patients were in the hyperkalemia group whereas 34 out of 2840 (1.20%) patients were in the control group. The mean eGFR of patients using multiple RAASi was significantly lower in the hyperkalemia than in the control group (46.3 ± 18.0 vs. 70.7 ± 22.8, mL/min/1.73 m^2^, *p*=0.028). These data indicated that advanced CKD patients using a combination of RAASi were at high risk of developing hyperkalemia.

### 3.2. The Association between Clinical Parameters and Serum Potassium Level

**Figure 2:**
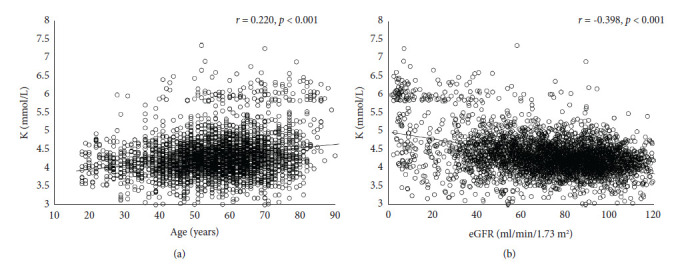
(a) The relationship between serum potassium level and age. The patient's age had a positive association with serum potassium level (*r* = 0.220, *p* < 0.001). The equation was as follows: *Y* = 0.010*X* + 3.788. (b) The relationship between serum potassium level and eGFR. Renal function assessed by eGFR had an inverse association with serum potassium level (*r* = −0.398, *p* < 0.001). The equation was as follows: *Y* = −0.008*X* + 4.935. eGFR, estimated glomerular filtration rate.

Serum potassium values from 2971 unique adult patients were used to correlate with the clinical data. The patient's age had a positive association with serum potassium level (*r* = 0.220, *p* < 0.001). The equation was as follows: *Y* = 0.010*X* + 3.788, where *Y* is the serum potassium level and *X* is the age of the patient (Figure 2(a)). Elderly patients generally had higher serum potassium than younger patients. In patients with hyperkalemia, more males were affected than females (*p*=0.022, Table 1).

**Figure 3:**
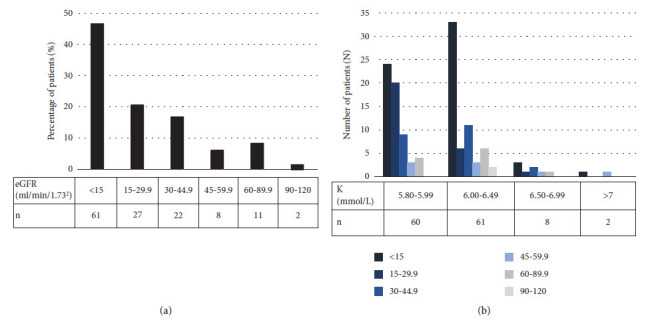
(a) The number and the percentage of patients with hyperkalemia at each CKD staging. (b) The number of patients in each CKD staging according to varying levels of high serum potassium. 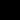 eGFR < 15 ml/min/1.73 m^2^, 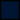 eGFR = 15–29.9 ml/min/1.73 m^2^, 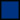 eGFR = 30–44.9 ml/min/1.73 m^2^, 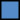 eGFR = 45–59.9 ml/min/1.73 m^2^, 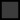 eGFR = 60–89.9 ml/min/1.73 m^2^, and 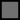 eGFR = 90–120 ml/min/1.73 m^2^. eGFR, estimated glomerular filtration rate; K, serum potassium.

Renal function (assessed by eGFR) had an inverse association with serum potassium level (*r* = −0.398, *p* < 0.001). The equation was as follows: *Y* = −0.008*X* + 4.935, where *Y* represents the serum potassium level and *X* represents the eGFR of the patient (Figure 2(b)). Using CKD staging in the hyperkalemia group revealed that advanced CKD patients were the majority of the population in this study (Table 1 and Figure 3(a)). From a different perspective, grouping patients by serum potassium levels also demonstrated that the high CKD staging patients were the majority of the population in every range of high serum potassium levels (Figure 3(b)).

**Figure 4:**
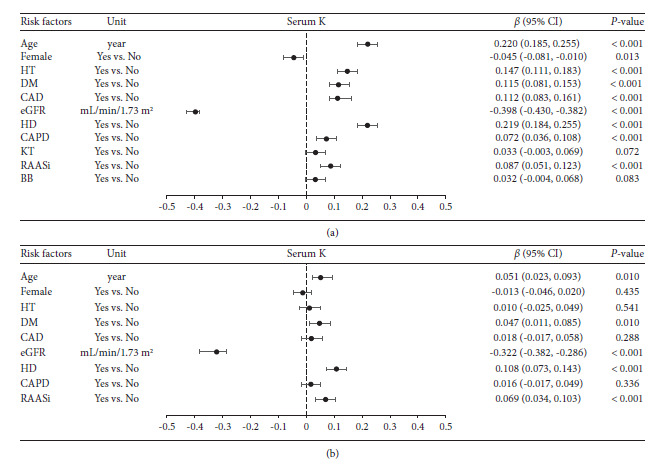
Baseline patient characteristics that were associated with serum potassium levels. Data were analyzed by univariate (a) and multivariate (b) linear regression analyses. K, potassium; HT, hypertension; DM, diabetes mellitus; CAD, coronary artery disease; eGFR, estimated glomerular filtration rate; HD, hemodialysis; CAPD, continuous ambulatory peritoneal dialysis; KT, kidney transplantation; RAASi, renin-angiotensin-aldosterone system inhibitor; BB, beta-blockers; *β*, standardized coefficient of *β*; CI, confidence interval for *β*.

By using univariate linear regression analysis, the study indicated that the serum potassium level was significantly associated with 7 clinical characteristics, including age (*r* = 0.220, *p* < 0.001), presence of hypertension (*r* = 0.147, *p* < 0.001), diabetes mellitus (*r* = 0.115, *p* < 0.001), CAD (*r* = 0.112, *p* < 0.001), hemodialysis treatment (*r* = 0.219, *p* < 0.001), CAPD treatment (*r* = 0.072, *p* < 0.001), and usage of RAASi (*r* = 0.087, *p* < 0.001) and inversely associated with 2 clinical characteristics, namely, female gender (*r* = −0.045, *p*=0.013) and eGFR (*r* = −0.398, *p* < 0.001) as shown in Figure 4(a).

A further study using multivariate linear regression analysis demonstrated that the serum potassium level was independently and significantly associated only with age (*r* = 0.051, *p*=0.010), diabetes mellitus (*r* = 0.047, *p*=0.010), hemodialysis treatment (*r* = 0.108, *p* < 0.001) and usage of RAASi (*r* = 0.069, *p* < 0.001) and independently, significantly, and inversely associated only with eGFR (*r* = −0.322, *p* < 0.001) as shown in Figure 4(b).

**Figure 5:**
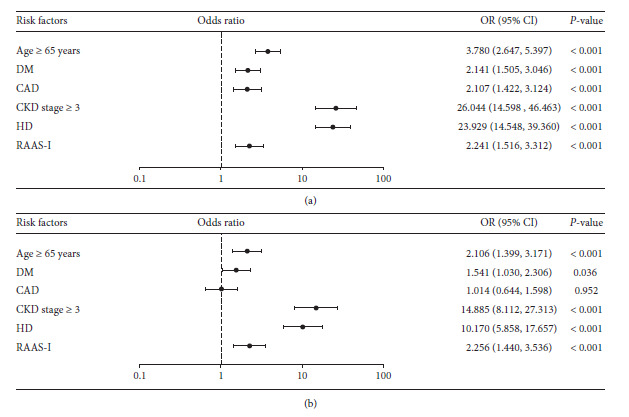
Baseline patient characteristics that were associated with the development of hyperkalemia (serum potassium ≥ 5.8 mmol/L). Data were analyzed by univariate (a) and multivariate (b) binary logistic regression to calculate the odds ratio. DM, diabetes mellitus; CAD, coronary heart disease; CKD, chronic kidney disease; HD, hemodialysis; RAASi, renin-angiotensin-aldosterone system inhibitor; OR; odds ratio; CI, confidence interval.

Using univariate binary logistic regression to calculate the odds ratio of serum potassium ≥5.8 mmol/L, the study indicated that the presence of hyperkalemia was significantly associated with 6 clinical characteristics, including age ≥65 years (odds ratio, 3.780; 95% CI, 2.647, 5.397; *p* < 0.001), presence of diabetes mellitus (odds ratio, 2.141; 95% CI, 1.505, 3.046; *p* < 0.001), CAD (odds ratio, 2.107; 95% CI, 1.422, 3.124; *p* < 0.001), CKD stage ≥3 (odds ratio, 26.044; 95% CI, 14.595, 46.463; *p* < 0.001), hemodialysis treatment (odds ratio, 23.292; 95% CI, 14.548, 39.360; *p* < 0.001), and usage of RAASi (odds ratio, 2.241; 95% CI, 1.516, 3.312; *p* < 0.001) as shown in Figure 5(b).

Further multivariate analysis revealed that the presence of hyperkalemia was independently and significantly associated with only 5 clinical characteristics, including age ≥65 years (odds ratio, 2.106; 95% CI, 1.399, 3.171; *p* < 0.001), presence of diabetes mellitus (odds ratio, 1.541; 95% CI, 1.030, 2.306; *p*=0.036), CKD stage ≥3 (odds ratio, 14.885; 95% CI, 8.112, 27.313; *p* < 0.001), hemodialysis treatment (odds ratio, 10.170; 95% CI, 5.858, 17.657; *p* < 0.001), and usage of RAASi (odds ratio, 2.256; 95% CI, 1.440, 3.536; *p* < 0.001) as shown in Figure 5(b).

## 4. Discussion

The prevalence of hyperkalemia in our study was 4.41%—this was conducted in the outpatient settings, department of medicine, where hyperkalemia was defined as serum potassium of more than or equal to 5.8 mmol/L. The prevalence of hyperkalemia can vary remarkably depending on the cut-off serum potassium level and the clinical setting of data collection (for example, inpatient or outpatient department). The estimation of the prevalence of hyperkalemia ranged from 1.1% to 10.0% among US hospitalized patients where hyperkalemia was defined as serum potassium of more than or equal to 6 mmol/L [19]. In a Canadian study on hospitalized elderly patients using 5.5 mmol/L as the cutoff for defining hyperkalemia, the prevalence of hyperkalemia was 2.6% to 3.5% [20]. The prevalence of hyperkalemia in Japanese hospitals was 6.79%, where hyperkalemia was defined as serum potassium of ≥5.1 mmol/L [21]. In a systematic review and meta-analyses (by any definition or threshold), the prevalence of adult hyperkalemia and hyperkalemia in the outpatient or primary care settings was 6.3% and 5%, respectively [16]. Our data were consistent with previous reports [16, 19–21].

In our study, the patient's age had an independent and positive association with serum potassium level (Figure 4(b)). Patients with hyperkalemia were, on average, 10 years older than the control group (66.5 vs. 55.9, *p* < 0.001). These results were in concordance with the previous report which demonstrated that age was an independent risk factor of hyperkalemia prevalence after adjusting for comorbidities [14]. Males had a higher risk of developing hyperkalemia in our study (51.1% vs. 40.7%), whereas the literature showed mixed findings [14, 22]. Renal function assessed by eGFR had an independent and inverse association with serum potassium level (Figure 4(b)). Advanced CKD patients were the majority of the population in this study (Figure 3(a)) which was in concordance with the previous report [16, 21]. In chronic kidney disease patients, the prevalence of hyperkalemia significantly increased from CKD stage 3B to stage 5 (Table 1 & Figure 3(a)).

Multivariate linear regression analysis indicated that the serum potassium level was independently and significantly associated with age, patients with diabetes mellitus, hemodialysis treatment, and usage of RAASi and independently, significantly, and inversely associated with eGFR. Further analysis using multivariate binary logistic regression revealed that hyperkalemia was independently and significantly associated with 5 clinical parameters, including age ≥65 years (odds ratio 2.106; *p* < 0.001), presence of diabetes mellitus (odds ratio 1.541; *p*=0.036), CKD stage ≥3 (odds ratio 14.885; *p* < 0.001), hemodialysis treatment (odds ratio 10.170; *p* < 0.001), and usage of RAASi (odds ratio 2.256; *p* < 0.001). These data indicated that patients with older age, diabetes mellitus, low eGFR, hemodialysis treatment, and usage of RAASi were at high risk of developing hyperkalemia in which CKD stage ≥3 was the highest risk factor among this group of patients. These risks would be further elevated if the patients had multiple risk factors or were on a combination of RAASi.

Approximately 98% of the total body potassium is accumulated in the intracellular compartment, with muscle comprising 80% of the intracellular potassium [23]. Although the primary control for potassium homeostasis is the renal excretion system, the regulation of extracellular potassium through taken up and released by skeletal muscles also plays an important role. It is well known that the uptake of potassium from the extracellular fluid by skeletal muscle is moderated mainly by the ubiquitous Na-K ATPase [24]. Alterations in the abundance of Na-K ATPase also may lead to interference with extracellular potassium homeostasis. Patients with diabetes mellitus or heart failure or the elderly have a reduced abundance of Na-K ATPase in skeletal muscle [25] and are more susceptible to drug-induced hyperkalemia as well as exercise-induced hyperkalemia [26, 27]. This phenomenon could explain the association between hyperkalemia and elderly patients and patients with diabetes mellitus.

In patients with low GFR (CKD), hemodialysis treatment, or usage of RAASi, hyperkalemia is likely to be caused by decreased renal excretion of potassium. Potassium homeostasis is achieved by balancing intake with excretion and appropriate distribution between intra- and extracellular fluid. The principal controls of potassium excretion are factors that regulate potassium secretion along the distal nephron such as mineralocorticoid activity, distal delivery of sodium, and luminal flow rate [2]. Aldosterone plays an important role in controlling potassium excretion at the collecting tubules of the kidneys. In the presence of hyperkalemia, the adrenal cortex stimulates the secretion of aldosterone into the bloodstream. The aldosterone acts on the kidneys, where it stimulates the secretion of potassium by the distal nephron, particularly the principal cells of the cortical collecting tubule, which results in increased potassium excretion [8]. Circulating aldosterone levels are the result of multiple controlling mechanisms, the most significant being those regulated by the renin-angiotensin system and plasma potassium levels. Another contributing factor is fluid volume; aldosterone plays a critical role in responding to volume depletion and hypotension. Taken together, aldosterone plays an important role in both regulating potassium homeostasis and the hemodynamics of circulatory volume in the body. The common causes of hyperkalemia from decreased renal excretion can be defined into 2 groups: (1) decrease in GFR such as CKD and (2) imperfect renal tubular secretion of potassium either from interference in the renin-angiotensin-aldosterone axis or tubular resistance to the action of aldosterone [13]. Hyperkalemia can be caused by CKD due to reduced renal mass and, consequently, a decrease in renal function (GFR) and potassium excretion that causes an accumulation of potassium in the body. On the other hand, drugs that interfere with the renin-angiotensin-aldosterone axis can also induce hyperkalemia by decreasing tubular potassium excretion. The usage of a combination of RAASi would intensify this effect and cause severe hyperkalemia. This is particularly important in CKD patients using multiple RAASi, who were at very high risk of developing hyperkalemia. In addition, some conditions that cause tubular resistance to the action of aldosterone (e.g., renal tubular acidosis and diabetes mellitus) can also cause hyperkalemia. In summary, disturbance in potassium excretion from low GFR or medications/conditions that interfere with renin-angiotensin-aldosterone activities would result in hyperkalemia.

Recommended medical treatment with RAASi has frequently been connected as a major facet in the development of hyperkalemia in patients with heart failure, CKD, or diabetes mellitus. The development of hyperkalemia in patients with diabetes mellitus, cardiac, or renal disease using RAASi creates a therapeutic conflict, as those patients who are at the highest risk for hyperkalemia are also the ones who would receive the greatest cardiovascular benefit from these medications [28, 29]. It emphasizes the strict monitoring of serum potassium levels required in these groups of patients to achieve the best therapeutic care.

## 5. Conclusions

The prevalence of hyperkalemia in our study was 4.41% in the outpatient setting. The patient's age had an independent and positive association with serum potassium level, while eGFR had an independent and inverse association. The risk factors of hyperkalemia were patients with age ≥65 years, presence of diabetes mellitus, CKD stage ≥3, hemodialysis treatment, and usage of RAASi. Patients who were at the highest risk of hyperkalemia were the same ones who were likely to gain the greatest cardiovascular benefit from these medications. This study highlighted the vital importance of monitoring serum potassium levels in these groups of patients.

### 5.1. Study Limitation

This study was a cross-sectional observational study. Therefore, all the data including clinical and laboratory tests might not be completely collected—approximately 8–10% of the data were missing. Also, this study was a single-center study, and it may not reflex hyperkalemia within the whole country's population. Our control population may not be the perfect control group as our hospital is a tertiary care center, and the majority of patients are likely to have some form of underlying medical condition. Another limitation of this study is that the patients were taken from the outpatient department of Medicine, so this is unlikely to reflect hyperkalemia that has been presented in other clinical settings. Further studies would be required in other clinical areas such as the emergency department, or inpatients, to provide a more comprehensive picture of the prevalence and risk factors contributing to hyperkalemia.

## Figures and Tables

**Table 1:** Baseline characteristics of patients with serum potassium ≥5.8 and <5.8 mmol/L in the 2021 calendar year.

	*K* ≥ 5.8 mmol/L (*N* = 131)	*K* < 5.8 mmol/L (*N* = 2840)	*p*-value
Age, years	66.5 ± 13.4	55.9 ± 12.9	<0.001
Female, *N* (%)	64 (48.9)	1683 (59.3)	0.022
Serum potassium, mmol/L	6.10 ± 0.28	4.24 ± 0.42	<0.001
GFR^*∗*^, CKD-EPI, mL/min/1.73 m^2^	16.9 (30.8)	78.3 (34.1)	<0.001
Hypertension, *N* (%)	91 (69.5)	1667 (58.7)	0.010
Diabetes, *N* (%)	72 (55.0)	1031 (36.3)	<0.001
Coronary artery diseases, *N* (%)	37 (28.2)	447 (15.7)	0.002
CKD stage ≥ 3, *N* (%)	118 (90.1)	734 (25.8)	<0.001
CKD stage 3A, *N* (%)	8 (6.1)	384 (13.5)	0.014
CKDI stage 3B, *N* (%)	22 (16.8)	227 (8.0)	<0.001
CKD stage 4, *N* (%)	27 (20.6)	61 (2.1)	<0.001
CKD stage 5, *N* (%)	61 (46.6)	62 (2.2)	<0.001
Hemodialysis, *N* (%)	34 (26.0)	41 (1.44)	<0.001
CAPD, *N* (%)	4 (3.05)	5 (0.18)	<0.001
Kidney transplantation, *N* (%)	7 (5.34)	41 (1.44)	0.001
Medication use			
RAASi, *N* (%)	38 (29.0)	438 (15.4)	0.001
ACE inhibitors, *N* (%)	7 (5.34)	113 (3.98)	0.438
ARB, *N* (%)	23 (17.6)	305 (10.7)	0.046
MRA, *N* (%)	13 (9.92)	57 (2.01)	0.003
Beta blockers, *N* (%)	60 (45.8)	1189 (41.9)	0.372
TMP/SMX, *N* (%)	6 (4.58)	65 (2.29)	0.219
Cyclosporine A/tacrolimus, *N* (%)	5 (3.82)	55 (1.94)	0.271

Data presented as mean ± SD or ^*∗*^median (IQR) or unless otherwise indicated. GFR, glomerular filtration rate; CKD-EPI, chronic kidney disease epidemiology collaboration; CAPD, continuous ambulatory peritoneal dialysis; RAASi, renin-angiotensin-aldosterone system inhibitor; ACE inhibitor, angiotensin-converting enzyme inhibitor; ARB, angiotensin receptor blocker; MRA, mineralocorticoid receptor antagonist; TMP/SMX, trimethoprim/sulfamethoxazole.

## Data Availability

The datasets generated and/or analyzed during the current study are not publicly available due to the institutional policy but are available from the corresponding author upon reasonable request.
